# Medical marijuana policies, opioid prescriptions, and adverse events among patients undergoing cancer resection surgery

**DOI:** 10.1002/cncr.70107

**Published:** 2025-10-01

**Authors:** Ju‐Chen Hu, Kenneth Karan, Hao Zhang, Russell Portenoy, William E. Rosa, Yiye Zhang, M. Carrington Reid, Rulla M. Tamimi, Fang Zhang, Eduardo Bruera, Judith A. Paice, Yuhua Bao

**Affiliations:** ^1^ Department of Health Policy and Management Celia Scott Weatherhead School of Public Health and Tropical Medicine Tulane University New Orleans Louisiana USA; ^2^ Department of Population Health Sciences Weill Cornell Medicine New York New York USA; ^3^ Department of Health Policy and Organization School of Public Health University of Alabama at Birmingham Birmingham Alabama USA; ^4^ Department of Family and Social Medicine Albert Einstein College of Medicine New York New York USA; ^5^ Department of Psychiatry & Behavioral Sciences Memorial Sloan Kettering Cancer Center New York New York USA; ^6^ Division of Geriatrics and Palliative Medicine Weill Cornell Medicine New York New York USA; ^7^ Department of Population Medicine Harvard Pilgrim Health Care Institute Boston Massachusetts USA; ^8^ Harvard Medical School Boston Massachusetts USA; ^9^ Department of Palliative Rehabilitation and Integrative Medicine University of Texas MD Anderson Cancer Center Houston Texas USA; ^10^ Division of Hematology‐Oncology Northwestern University Feinberg School of Medicine Chicago Illinois USA

**Keywords:** medical marijuana, cannabis, cancer, pain management, opioids

## Abstract

**Background:**

Opioid use and adverse events among cancer patients may change with access to medical marijuana. This study investigated the impacts of medical marijuana legalization (MML) since 2016.

**Methods:**

Using a difference‐in‐differences approach and 2016 to 2022 private insurance claims data, this cross‐sectional study included patients (aged 18–64 years) undergoing resection surgery for newly diagnosed (female) breast, colorectal, or lung cancer in 27 states without MML as of 2016. MML policies were classified into (1) no MML, (2) MML without dispensaries (after MML effective date and before the first state‐licensed dispensary opened), and (3) MML with dispensaries. Outcomes included during the 6 months postdiagnosis: (1) any opioid prescription, (2) any short‐acting oxycodone, hydrocodone, hydromorphone, or morphine prescription (“strong opioids”), (3) any short‐acting tramadol or codeine prescription (“weak opioids”), and (4) total morphine milligram equivalents among patients with opioid prescriptions, (5) any all‐cause, and (6) any pain‐related emergency department visits or hospitalizations.

**Results:**

The sample (*N* = 34,911) included 24,592 patients with breast, 8510 colorectal, and 1809 lung cancer. Compared to no MML, MML with dispensaries was associated with reduced any strong short‐acting opioids prescription use (difference = −4.6; 95% CI, −8.6 to −0.5 percentage points [pp]; *p* = .028) and increased any all‐cause adverse hospital events (difference = 2.6; 95% CI, 0.7−4.5 pp; *p* = .006). MML without dispensaries was associated with increased any weak opioid prescription use (difference = 1.2; 95% CI, 0.5−2 pp; *p* = .002).

**Conclusions:**

MML policies may have affected the type of opioid prescribed and increased adverse hospital events among patients with cancer and resection surgery. Additional investigation of medical marijuana’s impact on cancer pain management is warranted.

## Abstract 2

Many states have implemented medical marijuana legalization (MML) but the impacts of MML policies among patients undergoing cancer resection surgery remain underexplored. This study found that while MML policies, both with and without dispensaries, were not associated with significant changes in any opioid prescription use, they may have affected the type of opioids patients received and all‐cause emergency department visits and hospitalizations.

## INTRODUCTION

Although opioid therapy continues to be the mainstay approach for managing moderate‐to‐severe cancer pain,^1^ prescription opioid use among patients with cancer has declined substantially since 2016.^2^, ^3^ This decline has been accompanied by an evident need for alternative pain relief, reflected in part by the increased use of marijuana among patients with cancer.^4^, ^5^, ^6^ Notably, pain is the most commonly cited reason for medical marijuana use in the cancer population.^4^, ^7^, ^8^

The rapidly evolving landscape of medical marijuana legalization (MML) has important implications for patients with cancer. As of 2024, 38 states had legalized medical marijuana and all 38 states except Maryland included cancer as a qualifying condition for medical marijuana use.^9^, ^10^ MML policies vary in the extent to which they facilitate access to medical marijuana. One important factor is the availability of state‐licensed dispensaries.^11^ As of 2023, 34 of 38 states with MML had at least one state‐licensed medical dispensary.^12^ Notably, these dispensaries often did not open until several years after MML took effect in the state. This delay in dispensary access can influence whether patients choose to try medical marijuana, change their existing usage patterns, or seek marijuana from unregulated sources.

Despite its rising use among patients with cancer, evidence to recommend for or against medical marijuana in cancer‐related pain and symptom management remains insufficient.^13^, ^14^ Although clinicians generally believed that medical marijuana has a lower risk of misuse and overdose than opioids, concerns persisted regarding its effectiveness for cancer pain management and potential harms, including side effects, misuse, and drug–drug interactions affecting cancer treatments or other drug therapies, including opioids.^14^, ^15^, ^16^, ^17^ These potential harms may lead to adverse events, such as emergency department visits or hospitalizations. Moreover, the risk of harm may be heightened when patients use marijuana without consulting their health care providers, obtain it from unregulated sources, or use it without understanding the potential harm.

We sought to understand the potential impact of MML policies on patients with cancer during the years after 2016, when declines in prescription opioid use accelerated.^2^, ^3^ We focused on patients who were newly diagnosed with cancer and underwent cancer resection surgery—a subpopulation with prevalent pain management needs. A meta‐analysis study estimated that approximately 40% of patients with cancer undergoing resection surgery experienced a significant burden of pain,^18^ leading to the consideration of opioid therapies.^1^ We aim to understand how MML policies have affected pain management and outcomes among this population. Specifically, we examined the associations between MML, both with and without state‐licensed medical marijuana dispensaries, and opioid prescriptions and adverse hospital events experienced by these patients. We hypothesized that, at the population level, increased availability of medical marijuana (considered least available in states with no MML and most available in states with MML and state‐licensed dispensaries) would be associated with lower rates of opioid prescriptions and increased rates of adverse hospital events.

## METHODS

### Policies of interest: MML with and without dispensaries

We included 27 states that had not implemented MML as of January 1, 2016. Among states that implemented MML during 2016 through 2022, we identified the effective date of MML and the opening date of the first state‐licensed dispensary if the state had opened any dispensary by December 31, 2022 (Supporting Information S1: Appendix 1). These data were compiled from the National Conference of State Legislatures, the RAND‐USC Schaeffer Opioid Policy Tools and Information Center, the Marijuana Policy Project, and the Insurance Institute for Highway Safety.^12^, ^19^, ^20^, ^21^

A three‐category MML policy was created for analysis: (1) no MML, (2) MML without dispensaries, and (3) MML with dispensaries. From 2016 to 2022, among the 27 study states, 13 states never implemented MML; three states switched from no MML to MML without dispensaries; and 11 states switched from no MML to MML without dispensaries first and then to MML with dispensaries (Supporting Information S1: Appendix 1).

### Data and sample

We used the Health Care Cost Institute (HCCI) private insurance database, which covers about one‐third of privately insured individuals in the United States.^22^ Our study sample included individuals aged 18 to 64 who were newly diagnosed with one of three common cancers^23^—(female) breast, colorectal, or lung—and who underwent cancer resection surgery within 6 months of diagnosis.

To construct our sample, we identified the first primary diagnosis of (female) breast, colorectal, or lung cancer received by a patient between January 1, 2016, and July 31, 2022. We then used the month of the first primary cancer diagnosis as the index and established a 12‐month lookback period (before the diagnosis month) and a 6‐month observation period starting from the diagnosis month and required patients to be continuously enrolled in insurance over these 18 months. We restricted the sample to patients who received resection surgery for their primary cancer diagnosis during the 6‐month observation period (Supporting Information S1: Appendix 2). We excluded patients who received active cancer treatment (Supporting Information S1: Appendix 3) or opioid prescriptions during the 12‐month lookback period. We provide the sample derivation flowchart in Supporting Information S1: Appendix 4.

### Outcomes

We examined opioid prescriptions and adverse hospital events during the 6‐month observation period. Notably, to better reflect patient’s pain management approach during the postdiagnosis period, we examined these outcomes across the observation period rather than limiting to those specifically attributed to resection surgery. For opioid prescriptions, we used pharmacy claims to measure if the patient had at least an (1) opioid prescription, (2) a short‐acting opioid prescription typically used for moderate‐severe pain (including oxycodone, hydrocodone, hydromorphone, or morphine; hereafter “any strong short‐acting opioid prescription”), and (3) short‐acting tramadol or codeine prescription (consistent with usual clinical use for less severe pain; hereafter “any weak short‐acting opioid prescription”).^24^ We also measured (4) total morphine milligram equivalents (MMEs) received by patients with an opioid prescription. We used data published by the Centers for Disease Control and Prevention to identify the National Drug Codes and MME conversion factors for measuring total MMEs of opioid prescriptions.^25^ Buprenorphine formulations for treating opioid use disorder were excluded.^25^ For adverse hospital events, we measured if the patient had (5) any all‐cause emergency department (ED) visits or hospitalizations (excluding hospitalizations for cancer treatment, see Supporting Information S1: Appendix 3), and (6) any pain‐related ED visits or hospitalizations (Supporting Information S1: Appendix 5).^23^

### Statistical analysis

We applied a generalized difference‐in‐differences approach for analysis. The models included the three‐category measure of exposure to MML policy and a series of dichotomous indicators of calendar years to control for national secular trends and dichotomous indicators of states to control for between‐state differences in the outcome that did not change over time. The difference‐in‐differences approach relies on the assumption that, in the absence of the policy intervention, the outcome of interest would have followed a similar trend in states with and without the policy exposure (i.e., the “parallel trends assumption”). We conducted event study analysis to assess this assumption (Supporting Information S1: Appendices 6 and 7).

We estimated logistic models (i.e., generalized linear models with a logit link function and a binomial distribution) to examine dichotomous outcomes. To examine MMEs, we used generalized linear models with a log link function and gamma distribution of error terms based on the results of the modified Park test.^26^ We estimated robust standard errors recognizing individuals clustered within a state. All models controlled for patient sex, age, baseline comorbid conditions associated with needs for and/or risks of opioid analgesics (back pain, neck pain, arthritis and joint pain, other pain, mental health conditions, alcohol or drug use disorder, and nicotine dependence) during the 12‐month lookback period, and cancer treatment other than resection surgery during the 6‐month observation period (radiation therapy, chemotherapy, immunotherapy, and hormonal therapy; see Supporting Information S1: Appendix 3). Data on race and ethnicity were unavailable in HCCI. We also controlled for major policies addressing unsafe opioid prescriptions during study years, including state laws limiting the quantity or duration of opioids, and “must‐access” prescription drug monitoring programs that applied to all prescribers without allowing for prescriber discretion. Because only one of 27 study states legalized recreational marijuana by the end of 2022 (Missouri: December 8, 2022), we controlled for recreational marijuana legalization in our analysis and did not separately examine recreational marijuana as a policy of interest. We pooled samples from the three types of cancer for the main analysis and conducted secondary analysis by cancer type.

Analyses were performed using SAS version 9.4 and Stata SE version 17. For ease of interpretation, we used the “margins” command in Stata to report the marginal effects of MML policies (i.e., changes in predicted probability of dichotomous outcomes, and changes in MMEs).^27^ Results of all full regression models are presented in Supporting Information S1: Appendix 8.

Staggered policy implementation across states (as was the case in our study) may lead to potential biases in the difference‐in‐differences estimates because of heterogeneous policy effects.^28^, ^29^ For supplemental analysis, we applied the de Chaisemartin and d’Haultfoeuille model^28^ to assess the robustness of our results (Supporting Information S1: Appendix 9).

The study protocol is approved by the Weill Cornell Medicine institutional review board. We followed the STROBE reporting guidelines for cross‐sectional studies. Cells with <800 patients contributing to the estimates were suppressed to comply with the Health Care Cost Institute Reporting Guidelines.

## RESULTS

### Sample characteristics and unadjusted outcomes

Our sample (*N* = 34,911, 83.6% female) included 24,592 patients with (female) breast cancer, 8510 patients with colorectal cancer (41.9% female), and 1809 patients with lung cancer (55.8% female) (Table 1). About half (49.5%) of the patients were 55 to 64 years old. Non–cancer‐related pain was a common comorbid condition: 16.1% of patients had back pain; 23.0% had arthritis and joint pain; and 21.4% had other pain. About one‐fifth (21.6%) of patients had a mental health condition. About four‐fifths (79.28%) of patients received resection surgery within 60 days of their cancer diagnosis (Supporting Information S1: Appendix 10). In addition to cancer resection surgery, 41.6% of patients received radiation therapy and 42.3% received chemotherapy during the 6‐month observation period.

**TABLE 1:** Sample characteristics and unadjusted outcomes.

	All patients	
*N*	34,911	
Sample characteristics	*N*	%
Cancer type		
Breast	24,592	70.4
Colorectal	8510	24.4
Lung	1809	5.2
Female	29,169	83.6
Age		
18−44	5118	14.7
45−54	12,519	35.9
55−64	17,274	49.5
Comorbidity (during the 12‐month lookback period)		
Back pain	5613	16.1
Neck pain	2955	8.5
Arthritis and joint pain	8013	23.0
Other pain	7484	21.4
Mental health conditions	7542	21.6
Alcohol or drug use disorder	<800	n/a
Nicotine dependence	1294	3.7
Cancer treatment (during the 6‐month observation period since the new cancer diagnosis)		
Radiation therapy	14,527	41.6
Chemotherapy	14,769	42.3
Immunotherapy	3788	12.0
Hormonal therapy	<800	n/a
Unadjusted outcomes		
Opioid prescriptions		
Any opioid prescription	13,868	39.7
Any strong short‐acting opioid prescription (i.e., oxycodone, hydrocodone, hydromorphone, or morphine)	11,784	33.8
Any weak short‐acting opioid prescription (i.e., tramadol or codeine)	3638	10.4
Total MMEs if any opioid prescription, mean (SD)	414.62 (1261.83)	
Adverse hospital events		
All‐cause ED visits or hospitalizations (excluding cancer treatment)	7056	20.2
Pain‐related ED visits or hospitalizations	848	2.4

Cells with <800 patients contributing to the estimates were suppressed to comply with the Health Care Cost Institute Reporting Guidelines.

Abbreviations: ED, emergency department; MME, morphine milligram equivalent.

During the 6‐month observation period, 39.7% of patients had at least one opioid prescription; 33.8% received a strong short‐acting opioid prescription and 10.4% received a weak short‐acting opioid prescription (Table 1). Among patients with opioid prescriptions, the mean total MME was 414.6 (SD = 1261.83). About one‐fifth (20.2%) of patients had an ED visit or hospitalization that was not for cancer treatment and 2.4% had a pain‐related ED visit or hospitalization.

Results of sample characteristics and unadjusted outcomes stratified by cancer types are provided in Supporting Information S1: Appendix 11.

### MML policies and opioid prescriptions

**FIGURE 1:**
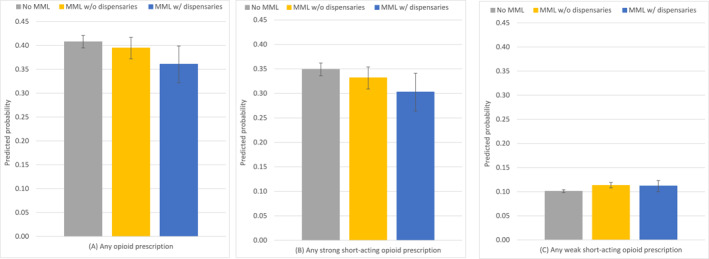
Predicted probabilities of opioid prescription outcomes associated with different medical marijuana policies. Whiskers span the 95% CI of estimates. MML indicates medical marijuana legalization.

While MML policies, both with and without dispensaries, were not associated with significant changes in the rate of any opioid prescriptions, we found that the associations differed by the type of opioid prescribed. Specifically, compared with no MML, MML with dispensaries was associated with a 4.6‐percentage‐point reduction (95% CI, −0.086 to −0.005; *p* = .028) in the rate of any strong short‐acting opioid prescription (no MML = 0.349; 95% CI, 0.338−0.360 vs MML with dispensaries = 0.303; 95% CI, 0.272−0.334; relative difference = −13.2%) (Figure 1, Table 2). Additionally, compared with no MML, MML without dispensaries was associated with a 1.2‐percentage‐point increase (95% CI, 0.005−0.020; *p* = .002) in the rate of any weak short‐acting opioid prescription (no MML = 0.101; 95% CI, 0.098−0.104 vs MML without dispensaries = 0.113; 95% CI, 0.108−0.119; relative difference = 11.9%) (Figure 1, Table 2).

**TABLE 2:** Estimates of the effects of medical marijuana policies on outcomes.

	MML w/o dispensaries (vs no MML)				MML w/dispensaries (vs no MML)				Incremental effects of dispensaries (MML w/dispensaries vs MML w/o dispensaries)			
Policy effect	Estimate	95% CI		*p*	Estimate	95% CI		*p*	Estimate	95% CI		*p*
Any opioid prescription	−0.013	−0.045	0.019	.438	−0.047	−0.098	0.004	.072	−0.034	−0.074	0.005	.088
Any strong short‐acting opioid prescription	−0.017	−0.046	0.012	.249	−0.046^a^	−0.086	−0.005	.028	−0.029	−0.067	0.010	.144
Any weak short‐acting opioid prescription	0.012^a^	0.005	0.02	.002	0.011	−0.003	0.025	.135	−0.001	−0.013	0.010	.812
Total MMEs if any opioid prescription	−51.72	−122.60	19.17	.153	−54.33	−155.48	46.81	.292	−2.61	−61.40	56.18	.931
All‐cause ED visits or hospitalizations	0.010	0.000	0.021	.050	0.026^a^	0.007	0.045	.006	0.016	−0.001	0.032	.061
Pain‐related ED visits or hospitalizations	0.007	−0.005	0.019	.241	0.01	−0.010	0.031	.315	0.004	−0.010	0.017	.612

Weak short‐acting opioids included tramadol and codeine. Strong short‐acting opioids included oxycodone, hydrocodone, hydromorphone, and morphine.

Abbreviations: ED, emergency department; MME, morphine milligram equivalent; MML, medical marijuana legalization.

^a^ indicates statistically significant results at 0.05 level.

When stratified by cancer type, we observed similar findings. Among patients with breast cancer, compared with no MML, MML with dispensaries was associated with a 4.8‐percentage‐point reduction (95% CI, −0.088 to −0.009; *p* = .016) in the rate of any strong short‐acting opioid prescription. MML without dispensaries was associated with a 1.6‐percentage‐point increase (95% CI, 0.001‐0.031; *p* = .035) in the rate of any weak short‐acting opioid prescription (Supporting Information S1: Appendices 12 and 13). While similar patterns were observed among colorectal and lung cancer patients, the associations did not reach statistical significance.

**FIGURE 2:**
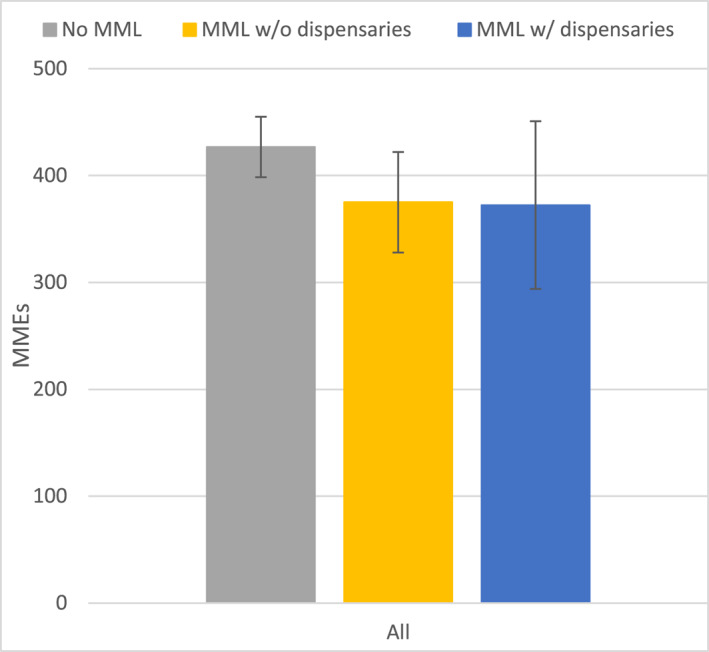
Estimated total MMEs associated with different medical marijuana policies among patients with any opioid prescription. Whiskers span the 95% CI of estimates. MME indicates morphine milligram equivalents.

Among patients with any opioid prescription, both MML with and without dispensaries were associated with lower total MMEs (vs no MML), but the differences were not statistically significant in the overall sample (Figure 2, Table 2). However, among colorectal cancer patients with any opioid prescription, MML without dispensaries was associated with a reduction of 181.26 (95% CI, −319.44 to −43.07; *p* = .01) in total MMEs when compared with no MML (Supporting Information S1: Appendices 12 and 14).

Supplemental analysis using the de Chaisemartin and d’Haultfoeuille method showed similar results in terms of directions, magnitude, and statistical significance with the main analysis (Supporting Information S1: Appendix 8).

### MML policies and adverse hospital events

**FIGURE 3:**
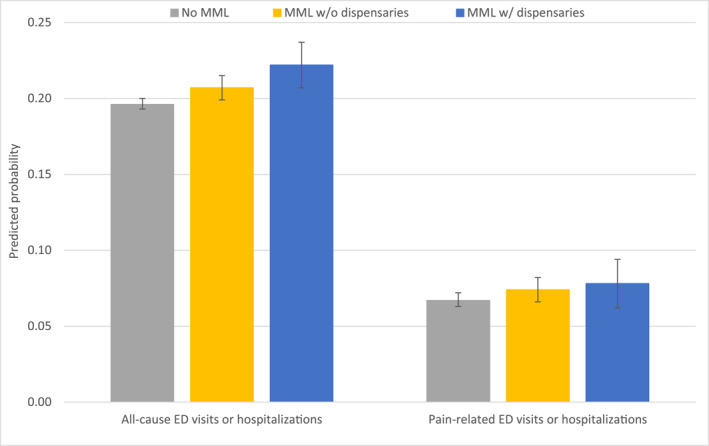
Predicted probabilities of adverse hospital events associated with different medical marijuana policies. Whiskers span the 95% CI of estimates. ED indicates emergency department; MML, medical marijuana legalization.

Compared with no MML, MML with dispensaries was associated with a 2.6‐percentage‐point increase (95% CI , 0.007−0.045; *p* = .006) in the rate of all‐cause ED visits or hospitalizations (no MML = 0.196; 95% CI, 0.193−0.200 vs MML with dispensaries = 0.222; 95% CI, 0.207−0.237; relative difference = 13.3%). MML without dispensaries was associated with a 1‐percentage‐point increase, compared to no MML (95% CI, 0.00−0.021; *p* = .05) in the same outcome (MML without dispensaries = 0.207; 95% CI, 0.199−0.215; relative difference to no MML = 5.6%) (Table 2, Figure 3).

Similar results were observed in stratified analysis by cancer type. For both patients with breast and colorectal cancer, MML with dispensaries (vs no MML) was associated with an increase in the rate of any all‐cause ED visits and hospitalizations (breast = 0.022; 95% CI, 0.001−0.043; *p* = .04; colorectal = 0.051, 95% CI , 0−0.102; *p* = .049) (Supporting Information S1: Appendices 12 and 15).

Neither MML without dispensaries nor MML with dispensaries was associated with meaningful changes in pain‐related ED visits or hospitalizations when compared to no MML (Table 2, Figure 3; Supporting Information S1: Appendices 12 and 15).

Our supplemental analysis using the de Chaisemartin and d’Haultfoeuille method showed similar results, although all estimates reached statistical significance (Supporting Information S1: Appendix 9).

## DISCUSSION

In this study of privately insured cancer patients undergoing resection surgery, we found that although MML policies were not associated with significant changes in the rate of any opioid prescriptions, they may have affected the type of opioids and MMEs patients received and adverse hospital events. Specifically, implementing MML with dispensaries, compared to no MML, was associated with a 13.2% relative reduction in the rate of any strong short‐acting opioid prescription and a 13.3% relative increase in the rate of any all‐cause adverse hospital events. Additionally, implementing MML without dispensaries was associated with an increased rate of any weak short‐acting opioid prescription among all patients and a reduction in total MMEs among patients with colorectal cancer with opioid prescriptions.

Our findings regarding opioid prescriptions suggest that the availability of medical marijuana under different MML policies may be a consideration in the clinical decision‐making applied to opioid prescribing for patients who received cancer resection surgery. Specifically, we found that while MML policies may not have affected whether patients received any opioid prescription, they may have affected the type and quantity of opioids they filled. Several mechanisms may explain these findings. First, when medical marijuana is legalized, patients may be more open to discussing the use of medical marijuana and disclosing their current and past marijuana use with their healthcare providers. A recent study of cancer patients in 12 cancer centers found that a higher proportion of patients in MML states discussed marijuana use with their providers than patients in states without MML.^30^ This increased openness may shift providers' decisions toward substituting weak opioids for strong opioids and/or prescribing opioids at lower doses or shorter duration. Second, some patients may perceive a lower need for opioids when medical marijuana is available and may choose not to fill their opioid prescriptions or to selectively fill the less potent opioids. Third, providers in states with MML may be more willing to act on their concerns about opioid toxicity or misuse with the belief that patients have more options for pain management. A study of gynecologic cancer patients in two academic centers in California and Colorado found that patients had decreased prescription opioid use after they started using medical marijuana.^31^

We found that MML with dispensaries was associated with increased adverse hospital events, highlighting potential safety concerns related to expanded access to medical marijuana. Many studies have reported side effects from marijuana use among patients with cancer, including drowsiness, hypotension, mental clouding, depression, lethargy, and nausea and vomiting.^32^, ^33^, ^34^ These side effects may be severe enough to result in ED visits or hospitalizations. Moreover, there is limited knowledge regarding potential drug–drug interactions between marijuana and cancer treatment and other medications, raising concerns about whether these unknown interactions could contribute to adverse outcomes. This is especially concerning in cases where patients use marijuana without the awareness or monitoring of their health care providers, use amounts beyond what their health care providers recommend, or live in states where marijuana products (often containing high THC concentrations) are underregulated. Although our measures of adverse events reflect extreme events, our findings highlight that MML, particularly in the presence of dispensaries, may be associated with significant increases in negative outcomes for patients with cancer. As more evidence emerges regarding marijuana use‐related side effects and drug–drug interactions, future studies should examine how MML policies affect these adverse events among patients with cancer.

As more states legalize medical marijuana and medical dispensaries become more available, our findings underscore the need for ongoing monitoring of medical marijuana use and the timely updating of clinical guidelines and regulations. First, states should consider leveraging existing infrastructure to track medical marijuana use. For example, New York requires dispensaries to report medical marijuana products sold to patients to the state prescription drug monitoring program registry.^35^ This approach allows clinicians, pharmacists, and dispensaries across the state to monitor patients’ medical marijuana use and improve state oversight of dispensaries' practices. Second, clinicians should follow the American Society of Clinical Oncology’s guidelines to foster “open, nonjudgmental communication” about medical marijuana use.^14^ A recent study found that most patients with cancer did not discuss marijuana with their health care providers, often because they were never asked.^36^ By initiating these discussions, providers can better understand the marijuana products patients are considering or using, allowing them to provide clinical supervision and adjust prescriptions as needed. Last, more research is urgently needed to guide clinical practice and training. Recent studies found that most medical schools did not provide any education on medical marijuana and most oncology fellows did not feel sufficiently informed to give marijuana‐related clinical recommendations, highlighting significant gaps in both training and research in oncology care.^37^, ^38^ Studies that directly examine medical marijuana use, pain relief, adverse drug effects, and functional outcomes should be prioritized to inform the development of evidence‐based guidelines and improve patient care.

## LIMITATIONS

We do not have data on patients’ use of medical marijuana and are thus limited in illuminating the mechanisms linking MML policies and opioid prescriptions/adverse hospital events. Similarly, data on opioid prescriptions administered during inpatient stays are not available in claims. However, because medical marijuana is not available during inpatient stays, we do not expect this limitation to significantly affect our findings. Although we controlled major state policies during study years bearing implications for opioid prescriptions and hospital events, these controls were not perfect; additional policies and developments in a state may have coincided with MML policies and potentially biasing our estimates. Additionally, because of limitations on the availability of dispensary data (e.g., total number, locations), we were unable to measure or assess the impact of accessibility of dispensaries, an area that warrants further research.

## CONCLUSIONS

We found that although MML policies, both with and without dispensaries, were not associated with changes in any opioid prescriptions, they were associated with changes in the type of opioid prescribed and all‐cause hospital adverse events among privately insured patients with cancer undergoing resection surgery. Our findings suggest that MML policies may influence pain‐related clinical decision‐making and may have unintended negative impacts on some patients who received cancer resection surgery.

## AUTHOR CONTRIBUTIONS

**Ju‐Chen Hu**: Conceptualization; Investigation; Writing—original draft; Methodology; Validation; Visualization; Writing—review & editing; Formal analysis; Data curation; and Resources. **Kenneth Karan**: Validation; Writing—review & editing; Resources; Investigation; and Data curation. **Hao Zhang**: Methodology; Validation; Writing—review & editing; Investigation; Resources; and Conceptualization. **Russell Portenoy**: Writing—review & editing; Validation; Resources; Investigation; and Conceptualization. **William E. Rosa**: Investigation; Writing—review & editing; Validation; Resources; and Conceptualization. **Yiye Zhang**: Investigation; Validation; Writing—review & editing; Resources; and Conceptualization. **M. Carrington Reid**: Investigation; Validation; Writing—review & editing; Resources; and Conceptualization. **Rulla M. Tamimi**: Investigation; Validation; Writing—review & editing; Resources; and Conceptualization. **Fang Zhang**: Investigation; Methodology; Validation; Writing—review & editing; Resources; and Conceptualization. **Eduardo Bruera**: Investigation; Validation; Writing—review & editing; Resources; and Conceptualization. **Judith A. Paice**: Investigation; Validation; Writing—review & editing; Resources; and Conceptualization. **Yuhua Bao**: Conceptualization; Investigation; Funding acquisition; Writing—original draft; Writing—review & editing; Methodology; Validation; Supervision; Resources; Project administration; Formal analysis; and Visualization.

## CONFLICT OF INTEREST STATEMENT

Ju‐Chen Hu reports support from the National Institute on Drug Abuse outside the submitted work. Russell Portenoy reports royalties for textbook editorship from Oxford University Press and royalties for writing topics from UpToDate outside the submitted work. William E. Rosa is partially supported by the National Cancer Institute Comprehensive Cancer Center Award (P30 CA008748) and the Robert Wood Johnson Foundation Harold Amos Medical Faculty Development Program and reports royalties from Oxford University Press, Jones & Bartlett Learning, and Springer Publishing outside the submitted work. Fang Zhang reports grants to their institution from Pfizer and GlaxoSmithKline plc outside the submitted work. Yuhua Bao reports support from the National Institute on Drug Abuse outside the submitted work and consulting payment from Stanford University and the Patient Centered Outcomes Research Institute. Other authors have nothing to disclose.

## Supporting information

Supplementary Material

Supplementary Material

## Data Availability

The data that support the findings of this study are available from The Health Care Cost Institute. Restrictions apply to the availability of these data, which were used under license for this study. Data are available from https://healthcostinstitute.org/ with the permission of The Health Care Cost Institute.
