# Supplementary material for: Medical marijuana policies, opioid prescriptions, and adverse events among patients undergoing cancer resection surgery
Source: Cancer. 2025 Oct 1;131(19):e70107. doi: 10.1002/cncr.70107 (PMC12486740; doi:10.1002/cncr.70107)
Supplement: Supplementary file 1 — Supplementary Material [file CNCR-131-e70107-s002.docx]

**Supplementary Online Content**

**Medical Marijuana Policies, Opioid Prescriptions, and Adverse Events among Patients Undergoing Cancer Resection Surgery**

Appendix 1. Implementation timeline of medical marijuana policies in 27 study states

Appendix 2. Codes for identifying breast, colorectal, and lung cancer and their associated resection surgeries

Appendix 3. Codes for identifying active cancer treatment

Appendix 4. Sample derivation flowchart

Appendix 5. ICD‐9 and ICD‐10 diagnostic codes for identifying pain‐related emergency department visits and hospitalizations

Appendix 6. Event study analysis assessing the parallel trends assumption between states that implemented MML without dispensaries and those that did not yet implement among 27 study states

Appendix 7. Event study analysis assessing the parallel trends assumption between states that implemented MML with dispensaries and those that did not yet implement among 27 study states

Appendix 8. Full regression results of main analysis

Appendix 9. de Chaisemartin and d’Haultfoeuille difference-in-differences model and supplemental analysis results

Appendix 10. Distribution of days from cancer diagnosis to resection surgery

Appendix 11. Sample characteristics and unadjusted outcomes by cancer type

Appendix 12. Estimates of medical marijuana policy effects on outcomes by cancer type

Appendix 13. Predicted probabilities of opioid prescription outcomes associated with different medical marijuana policies by cancer type

Appendix 14. Estimated total MMEs associated with different medical marijuana policies among patients any opioid prescription by cancer type

Appendix 15. Predicted probabilities of adverse hospital events associated with different medical marijuana policies by cancer type

**Appendix 1. Implementation timeline of medical marijuana policies in 27 study states**

| **State** | **Effective date of MML** | **Open date of the first state-licensed medical marijuana dispensary** |
| --- | --- | --- |
| AL | 5/17/2021 |  |
| AR | 11/9/2016 | 5/11/2019 |
| FL | 1/3/2017 | 12/19/2018 |
| GA |  |  |
| ID |  |  |
| IN |  |  |
| IA |  |  |
| KS |  |  |
| KY | 1/1/2025 |  |
| LA | 5/19/2016 | 8/6/2019 |
| MS | 2/2/2022 |  |
| MO | 12/6/2018 | 10/17/2020 |
| NE |  |  |
| NC |  |  |
| ND | 4/18/2017 | 3/1/2019 |
| OH | 9/8/2016 | 1/16/2019 |
| OK | 8/25/2018 | 10/26/2018 |
| PA | 5/17/2016 | 2/15/2018 |
| SC |  |  |
| SD | 7/1/2021 | 7/1/2022 |
| TN |  |  |
| TX |  |  |
| UT | 12/3/2018 | 3/2/2020 |
| VA | 6/1/2020 | 10/17/2020 |
| WV | 4/19/2017 |  |
| WI |  |  |
| WY |  |  |

Note: MML=Medical Marijuana Legalization. No MML is defined as the period before the effective date of MML. MML without dispensaries is defined as the period between the effective date of MML and the open date of the first state-licensed medical marijuana dispensary. MML with dispensaries is defined as the period after the open date of the first state-licensed medical marijuana dispensary.

**Appendix 2. Codes for identifying breast, colorectal, and lung cancer and their associated resection surgeries**

|  | **ICD-10 diagnosis codes for identifying cancer diagnosis** | **Procedure codes for identifying resection surgery** | | |
| --- | --- | --- | --- | --- |
|  |  | **ICD-9** | **ICD-10** | **CPT** |
| **Breast** | C50 C500 C5001 C50011 C50012 C50019 C5002 C50021 C50022 C50029 C501 C5011 C50111 C50112 C50119 C5012 C50121 C50122 C50129 C502 C5021 C50211 C50212 C50219 C5022 C50221 C50222 C50229 C503 C5031 C50311 C50312 C50319 C5032 C50321 C50322 C50329 C504 C5041 C50411 C50412 C50419 C5042 C50421 C50422 C50429 C505 C5051 C50511 C50512 C50519 C5052 C50521 C50522 C50529 C506 C5061 C50611 C50612 C50619 C5062 C50621 C50622 C50629 C508 C5081 C50811 C50812 C50819 C5082 C50821 C50822 C50829 C509 C5091 C50911 C50912 C50919 C5092 C50921 C50922 C50929 | 8520‑8523  8525‑8525  8540‑8548 | 07T50ZZ 07T60ZZ 07T70ZZ 07T80ZZ 07T90ZZ 0H5T0ZZ 0H5T3ZZ 0H5T7ZZ 0H5T8ZZ 0H5U0ZZ 0H5U3ZZ 0H5U7ZZ 0H5U8ZZ 0H5V0ZZ 0H5V3ZZ 0H5V7ZZ 0H5V8ZZ 0H5W0ZZ 0H5W3ZZ 0H5W7ZZ 0H5W8ZZ 0H5WXZZ 0H5X0ZZ 0H5X3ZZ 0H5X7ZZ 0H5X8ZZ 0H5XXZZ 0HBT0ZZ 0HBT3ZZ 0HBT7ZZ 0HBT8ZZ 0HBU0ZZ 0HBU3ZZ 0HBU7ZZ 0HBU8ZZ 0HBV0ZZ 0HBV3ZZ 0HBV7ZZ 0HBV8ZZ 0HBW0ZZ 0HBW3ZZ 0HBW7ZZ 0HBW8ZZ 0HBWXZZ 0HBX0ZZ 0HBX3ZZ 0HBX7ZZ 0HBX8ZZ 0HBXXZZ 0HTT0ZZ 0HTU0ZZ 0HTV0ZZ 0HTWXZZ 0HTXXZZ 0KTH0ZZ 0KTJ0ZZ HBU8ZZ | 19301 19302 19303 19304 19305 19306 19307 |
| **Colorectal** | C18 C180 C181 C182 C183 C184 C185 C186 C187 C188 C189 C19 C20 | 1731-1739  4571-4579  4581-4583  4610-4613  4840-4869 | 0D1N0Z4 0D1N4Z4 0DBE0ZZ 0DBE3ZZ 0DBE4ZZ 0DBE7ZZ 0DBE8ZZ 0DBGFZZ 0DBLFZZ 0DBMFZZ 0DBNFZZ 0DBP0ZZ 0DBP4ZZ 0DTE0ZZ 0DTE4ZZ 0DTE7ZZ 0DTE8ZZ 0DTF0ZZ 0DTF4ZZ 0DTF7ZZ 0DTF8ZZ 0DTG0ZZ 0DTG4ZZ 0DTG7ZZ 0DTG8ZZ 0DTGFZZ 0DTH0ZZ 0DTH4ZZ 0DTH7ZZ 0DTH8ZZ 0DTK0ZZ 0DTL0ZZ 0DTL4ZZ 0DTL7ZZ 0DTL8ZZ 0DTLFZZ 0DTMFZZ 0DTN0ZZ 0DTN4ZZ 0DTN7ZZ 0DTN8ZZ 0DTNFZZ 0DTP0ZZ 0DTP4ZZ 0DTP7ZZ 0DTP8ZZ | 44140‑44160  44204‑44213  44395  44397  45110‑45123  45395  45397 |
| **Lung** | C34 C340 C3400 C3401 C3402 C341 C3410 C3411 C3412 C342 C343 C3430 C3431 C3432 C348 C3480 C3481 C3482 C349 C3490 C3491 C3492 | 3230-3259 | 0BBC4ZZ 0BBD4ZZ 0BBF4ZZ 0BBG4ZZ 0BBH4ZZ 0BBJ4ZZ 0BBK0ZZ 0BBK3ZZ 0BBK4ZZ 0BBK7ZZ 0BBL0ZZ 0BBL3ZZ 0BBL4ZZ 0BBL7ZZ 0BTC0ZZ 0BTC4ZZ 0BTD0ZZ 0BTD4ZZ 0BTF0ZZ 0BTF4ZZ 0BTG0ZZ 0BTG4ZZ 0BTH4ZZ 0BTJ0ZZ 0BTJ4ZZ 0BTK0ZZ 0BTK4ZZ 0BTL0ZZ 0BTL4ZZ 0BTM0ZZ 0BTM4ZZ | 32440‑32445  32480‑32491  32663 |

**Appendix 3. Codes for identifying active cancer treatment**

We provided the list of codes for identifying resection surgery, radiation therapy, chemotherapy, immunotherapy, and hormonal therapy in the supplemental file “**Appx data_Cancer treatment codes**.” This file includes the following two tabs:

- **DataDict:** A data dictionary that includes descriptions of each variable and label.
- **CanTrt:** A list of codes for identifying active cancer treatment. For each type of cancer treatment, we included all relevant ICD-9 Diagnosis Codes (label: Dx09), ICD-10 Diagnosis Codes (label: Dx10), ICD-9 Procedure Codes (label: Tx09), ICD-10 Procedure Code (label: Tx10), CPT Codes (label: TxCp), HCPCS codes (label: TxHc), National Drug Codes (label: NDC), and Revenue Center Codes (label: Rev).

These codes are compiled from the following references:

1. National Cancer Institute. Procedure Codes for SEER-Medicare Analyses. <https://healthcaredelivery.cancer.gov/seermedicare/considerations/procedure_codes.html>
2. National Cancer Institute: CanMED: NDC. <https://seer.cancer.gov/oncologytoolbox/canmed/ndconc/>
3. National Cancer Institute: CanMED: HCPCS. <https://seer.cancer.gov/oncologytoolbox/canmed/hcpcs/>
4. Warren JL, Parsons HM, Mariotto AB, Boyd E, Enewold L. Evaluation of the Completeness of Managed Care Data to Identify Cancer Diagnoses and Treatments for Patients in the SEER-Medicare Data. Med Care. 2023 Dec 1;61(12):846-857. doi: 10.1097/MLR.0000000000001936.
5. Jafari MD, Jafari F, Halabi WJ, Nguyen VQ, Pigazzi A, Carmichael JC, Mills SD, Stamos MJ. Colorectal Cancer Resections in the Aging US Population: A Trend Toward Decreasing Rates and Improved Outcomes. JAMA Surg. 2014 Jun;149(6):557-64. doi: 10.1001/jamasurg.2013.4930.
6. Aquina CT, Blumberg N, Probst CP, Becerra AZ, Hensley BJ, Noyes K, Monson JR, Fleming FJ. Large Variation in Blood Transfusion Use After Colorectal Resection: A Call to Action. Dis Colon Rectum. 2016 May;59(5):411-8. doi: 10.1097/DCR.0000000000000588.
7. Schonberg MA, Marcantonio ER, Li D, Silliman RA, Ngo L, McCarthy EP. Breast cancer among the oldest old: tumor characteristics, treatment choices, and survival. J Clin Oncol. 2010 Apr 20;28(12):2038-45. doi: 10.1200/JCO.2009.25.9796.
8. LaPar DJ, Nagji AS, Bhamidipati CM, Kozower BD, Lau CL, Ailawadi G, Jones DR. Seasonal variation influences outcomes following lung cancer resections. Eur J Cardiothorac Surg. 2011 Jul;40(1):83-90. doi: 10.1016/j.ejcts.2010.11.023.
9. Colla CH, Morden NE, Skinner JS, Hoverman JR, Meara E. Impact of payment reform on chemotherapy at the end of life. J Oncol Pract. 2012 May;8(3 Suppl):e6s-e13s. doi: 10.1200/JOP.2012.000539.
10. Noone AM, Lund JL, Mariotto A, Cronin K, McNeel T, Deapen D, Warren JL. Comparison of SEER Treatment Data With Medicare Claims. Med Care. 2016 Sep;54(9):e55-64. doi: 10.1097/MLR.0000000000000073.

**Appendix 4. Sample derivation flowchart**

**Analytic sample (total N=34,911)**

**Non-elderly adult, newly diagnosed, opioid naïve patients undergoing cancer resection surgery with continuous enrollment and no prior cancer treatment in 27 study states**

Breast: 24,592

Colorectal: 8,510

Lung: 1,809

**Patients with new cancer diagnosis**

Breast: 370,552

Colorectal: 108,243

Lung: 61,774

**Newly diagnosed cancer patients with 12+6 month continuous enrollment**

Breast: 119,617

Colorectal: 47,014

Lung: 29,829

**Non-elderly adult, newly diagnosed cancer patients with continuous enrollment in 27 study states**

Breast: 50,564

Colorectal: 20,038

Lung: 9,758

**Non-elderly adult, newly diagnosed patients undergoing cancer resection surgery with continuous enrollment in 27 study states**

Breast: 29,442

Colorectal: 10,463

Lung: 2,603

Requiring continuous enrollment during the 12-month lookback period (before the month of cancer diagnosis) and the 6-month observation period (starting from the month of cancer diagnosis)

Requiring patients to be 18-64 year-old and in the 27 study states

Requiring patients to receive resection surgery of the newly diagnosed cancer during the 6-month observation period

Requiring patients to have no opioid prescription and any cancer treatment during the 12-month lookback period

**Appendix 5. ICD‐9 and ICD‐10 diagnostic codes for identifying pain‐related emergency department visits and hospitalizations**

| **ICD-9 Code** | **ICD-9 Descriptor** | **ICD-10Code** | **ICD-10 Descriptor** |
| --- | --- | --- | --- |
| 338.29 | Other chronic pain | G89.29 | Other chronic pain |
| 338.3 | Neoplasm related pain (acute) (chronic) | G89.3 | Neoplasm related pain (acute) (chronic) |
| 379.91 | Pain in or around eye | H57.10 | Ocular pain, unspecified eye |
| 379.91 | Pain in or around eye | H57.11 | Ocular pain, right eye |
| 379.91 | Pain in or around eye | H57.12 | Ocular pain, left eye |
| 379.91 | Pain in or around eye | H57.13 | Ocular pain, bilateral |
| 388.70 | Otalgia, unspecified | H92.01 | Otalgia, right ear |
| 388.70 | Otalgia, unspecified | H92.02 | Otalgia, left ear |
| 388.70 | Otalgia, unspecified | H92.03 | Otalgia, bilateral |
| 388.70 | Otalgia, unspecified | H92.09 | Otalgia, unspecified ear |
| 388.71 | Otogenic pain | H92.09 | Otalgia, unspecified ear |
| 388.72 | Referred otogenic pain | H92.09 | Otalgia, unspecified ear |
| 528.01 | Oral Mucositis | K12.30 | Oral Mucositis (ulcerative), unspecified |
| 528.01 | Oral Mucositis | K12.31 | Oral Mucositis (ulcerative) due to antineoplastic therapy |
| 528.02 | Oral Mucositis | K12.32 | Oral Mucositis (ulcerative) due to other drugs |
| 528.09 | Oral Mucositis | K12.39 | Other Oral Mucositis (ulcerative) |
| 528.9 | Other and Unspecified Diseases of the Oral Soft Tissue | K13.79 | Other lesions of oral mucosa |
| 569.42 | Anal or rectal pain | K62.89 | Other specified diseases of anus and rectum |
| 719.40 | Pain in joint, site unspecified | M25.50 | Pain in unspecified joint |
| 719.48 | Pain in joint, other specified sites | M25.50 | Pain in unspecified joint |
| 719.49 | Pain in joint, multiple sites | M25.50 | Pain in unspecified joint |
| 719.41 | Pain in joint, shoulder region | M25.511 | Pain in right shoulder |
| 719.41 | Pain in joint, shoulder region | M25.512 | Pain in left shoulder |
| 719.41 | Pain in joint, shoulder region | M25.519 | Pain in unspecified shoulder |
| 719.42 | Pain in joint, upper arm | M25.521 | Pain in right elbow |
| 719.42 | Pain in joint, upper arm | M25.522 | Pain in left elbow |
| 719.42 | Pain in joint, upper arm | M25.529 | Pain in unspecified elbow |
| 719.43 | Pain in joint, forearm | M25.531 | Pain in right wrist |
| 719.43 | Pain in joint, forearm | M25.532 | Pain in left wrist |
| 719.43 | Pain in joint, forearm | M25.539 | Pain in unspecified wrist |
| 719.44 | Pain in joint, hand | M25.541 | Pain in joints of right hand |
| 719.44 | Pain in joint, hand | M25.542 | Pain in joints of left hand |
| 719.45 | Pain in joint, pelvic region and thigh | M25.551 | Pain in right hip |
| 719.45 | Pain in joint, pelvic region and thigh | M25.552 | Pain in left hip |
| 719.45 | Pain in joint, pelvic region and thigh | M25.559 | Pain in unspecified hip |
| 719.46 | Pain in joint, lower leg | M25.561 | Pain in right knee |
| 719.46 | Pain in joint, lower leg | M25.562 | Pain in left knee |
| 719.46 | Pain in joint, lower leg | M25.569 | Pain in unspecified knee |
| 719.47 | Pain in joint, ankle and foot | M25.571 | Pain in right ankle and joints of right foot |
| 719.47 | Pain in joint, ankle and foot | M25.572 | Pain in left ankle and joints of left foot |
| 719.47 | Pain in joint, ankle and foot | M25.579 | Pain in unspecified ankle and joints of unspecified foot |
| 723.8 | Other syndromes affecting cervical region | M53.81 | Other specified dorsopathies, occipito-atlanto-axial region |
| 723.8 | Other syndromes affecting cervical region | M53.82 | Other specified dorsopathies, cervical region |
| 723.8 | Other syndromes affecting cervical region | M53.83 | Other specified dorsopathies, cervicothoracic region |
| 724.2 | Lumbago | M54.5 | Low back pain |
| 724.1 | Pain in thoracic spine | M54.6 | Pain in thoracic spine |
| 723.8 | Other syndromes affecting cervical region | M54.81 | Occipital neuralgia |
| 724.5 | Backache, unspecified | M54.89 | Other dorsalgia |
| 724.5 | Backache, unspecified | M54.9 | Dorsalgia, unspecified |
| 729.1 | Myalgia and myositis, unspecified | M79.10 | Myalgia, unspecified site |
| 729.1 | Myalgia and myositis, unspecified | M79.11 | Myalgia of mastication muscle |
| 729.1 | Myalgia and myositis, unspecified | M79.12 | Myalgia of auxiliary muscles, head and neck |
| 729.1 | Myalgia and myositis, unspecified | M79.18 | Myalgia, other site |
| 729.5 | Pain in limb | M79.601 | Pain in right arm |
| 729.5 | Pain in limb | M79.602 | Pain in left arm |
| 729.5 | Pain in limb | M79.603 | Pain in arm, unspecified |
| 729.5 | Pain in limb | M79.604 | Pain in right leg |
| 729.5 | Pain in limb | M79.605 | Pain in left leg |
| 729.5 | Pain in limb | M79.606 | Pain in leg, unspecified |
| 729.5 | Pain in limb | M79.609 | Pain in unspecified limb |
| 729.5 | Pain in limb | M79.621 | Pain in right upper arm |
| 729.5 | Pain in limb | M79.622 | Pain in left upper arm |
| 729.5 | Pain in limb | M79.629 | Pain in unspecified upper arm |
| 729.5 | Pain in limb | M79.631 | Pain in right forearm |
| 729.5 | Pain in limb | M79.632 | Pain in left forearm |
| 729.5 | Pain in limb | M79.639 | Pain in unspecified forearm |
| 729.5 | Pain in limb | M79.641 | Pain in right hand |
| 729.5 | Pain in limb | M79.642 | Pain in left hand |
| 719.44 | Pain in joint, hand | M79.643 | Pain in unspecified hand |
| 729.5 | Pain in limb | M79.643 | Pain in unspecified hand |
| 729.5 | Pain in limb | M79.644 | Pain in right finger(s) |
| 729.5 | Pain in limb | M79.645 | Pain in left finger(s) |
| 719.44 | Pain in joint, hand | M79.646 | Pain in unspecified finger(s) |
| 729.5 | Pain in limb | M79.646 | Pain in unspecified finger(s) |
| 729.5 | Pain in limb | M79.651 | Pain in right thigh |
| 729.5 | Pain in limb | M79.652 | Pain in left thigh |
| 729.5 | Pain in limb | M79.659 | Pain in unspecified thigh |
| 729.5 | Pain in limb | M79.661 | Pain in right lower leg |
| 729.5 | Pain in limb | M79.662 | Pain in left lower leg |
| 729.5 | Pain in limb | M79.669 | Pain in unspecified lower leg |
| 729.5 | Pain in limb | M79.671 | Pain in right foot |
| 729.5 | Pain in limb | M79.672 | Pain in left foot |
| 729.5 | Pain in limb | M79.673 | Pain in unspecified foot |
| 729.5 | Pain in limb | M79.674 | Pain in right toe(s) |
| 729.5 | Pain in limb | M79.675 | Pain in left toe(s) |
| 729.5 | Pain in limb | M79.676 | Pain in unspecified toe(s) |
| 784.1 | Throat pain | R07.0 | Pain in throat |
| 786.52 | Painful respiration | R07.1 | Chest pain on breathing |
| 786.51 | Precordial pain | R07.2 | Precordial pain |
| 786.52 | Painful respiration | R07.81 | Pleurodynia |
| 786.59 | Other chest pain | R07.82 | Intercostal pain |
| 786.59 | Other chest pain | R07.89 | Other chest pain |
| 786.50 | Unspecified chest pain | R07.9 | Chest pain, unspecified |
| 789.00 | Abdominal pain, unspecified site | R10.0 | Acute abdomen |
| 789.09 | Abdominal pain, other specified site | R10.10 | Upper abdominal pain, unspecified |
| 789.01 | Abdominal pain, right upper quadrant | R10.11 | Right upper quadrant pain |
| 789.02 | Abdominal pain, left upper quadrant | R10.12 | Left upper quadrant pain |
| 789.06 | Abdominal pain, epigastric | R10.13 | Epigastric pain |
| 608.9 | Unspecified disorder of male genital organs | R10.2 | Pelvic and perineal pain |
| 625.9 | Unspecified symptom associated with female genital organs | R10.2 | Pelvic and perineal pain |
| 789.09 | Abdominal pain, other specified site | R10.2 | Pelvic and perineal pain |
| 789.09 | Abdominal pain, other specified site | R10.30 | Lower abdominal pain, unspecified |
| 789.03 | Abdominal pain, right lower quadrant | R10.31 | Right lower quadrant pain |
| 789.04 | Abdominal pain, left lower quadrant | R10.32 | Left lower quadrant pain |
| 789.05 | Abdominal pain, periumbilic | R10.33 | Periumbilical pain |
| 789.07 | Abdominal pain, generalized | R10.84 | Generalized abdominal pain |
| 789.00 | Abdominal pain, unspecified site | R10.9 | Unspecified abdominal pain |
| 784.0 | Headache | R51 | Headache |
| 338.19 | Other acute pain | R52 | Pain, unspecified |
| 780.96 | Generalized pain | R52 | Pain, unspecified |
| 784.92 | Jaw pain | R68.84 | Jaw pain |

Source: CMS Chemotherapy Measure (OP‐35), available at: https://qualitynet.cms.gov/outpatient/measures/chemotherapy/methodology

**Appendix 6. Event study analysis assessing the parallel trends assumption between states that implemented MML without dispensaries and those that did not yet implement among 27 study states**

Each figure below shows the estimated difference (and 95% confidence interval) between states that implemented MML without dispensaries and no MML states in half-year intervals before and after implementation, holding the difference in the quarter immediately before implementation (i.e., “-1” in the figure) at 0. Overall, during the pre-implementation periods (i.e., -8 to -1), almost all estimated differences were not statistically significantly different from zero, and there were no notable trends. These data do not show violations of the parallel trend assumption in the difference-in-differences method.

Notably, during post-implementation periods (i.e, 0 to 12), the decrease in any opioid prescription and any strong short-acting opioid prescription became more salient after the third half-year interval. In contrast, the increase in all-cause emergency department visits or hospitalizations emerged earlier in the first to fourth half-year intervals.


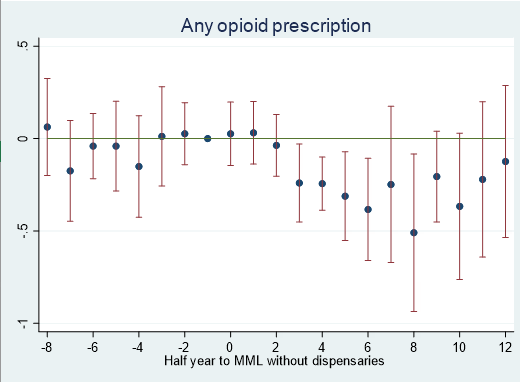

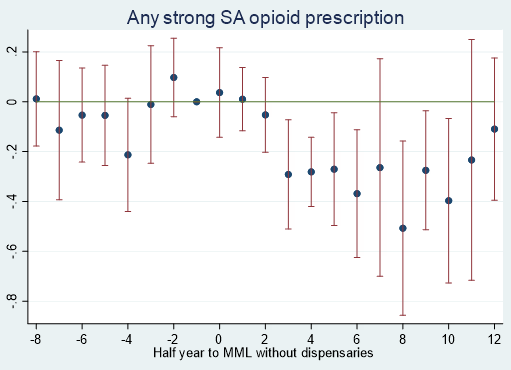

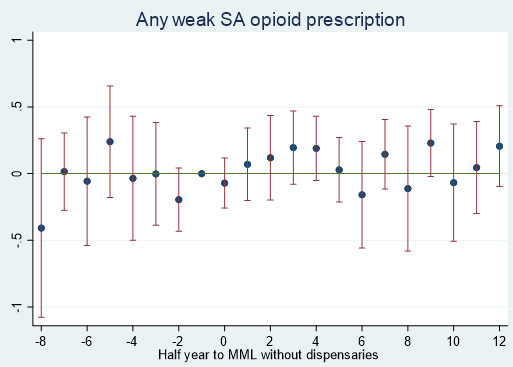


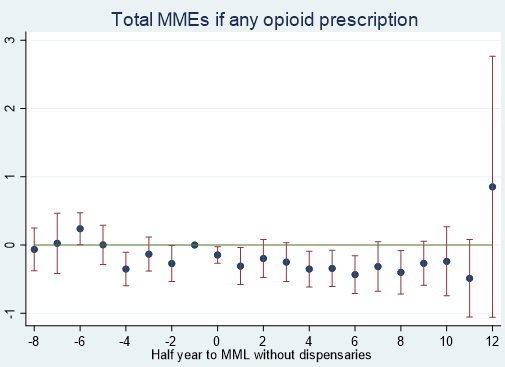

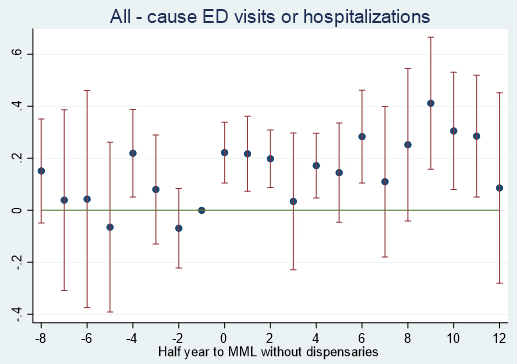

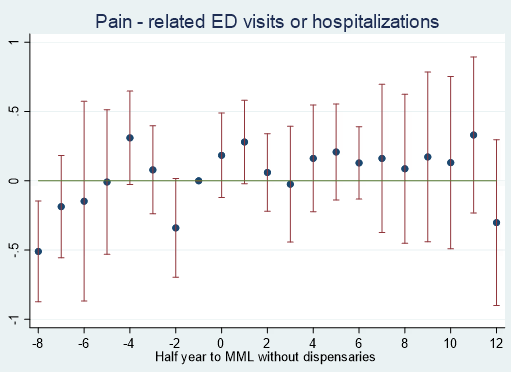


Note: MML=Medical Marijuana Legalization; SA=short-acting; MME=morphine milligram equivalent; ED=emergency department. Strong SA opioids included oxycodone, hydrocodone, hydromorphone, and morphine. Weak SA opioids included tramadol and codeine.

**Appendix 7. Event study analysis assessing the parallel trends assumption between states that implemented MML with dispensaries and those that did not yet implement among 27 study states**

Each figure below shows the estimated difference (and 95% confidence interval) between states that implemented MML with dispensaries and states that did not yet implement in half-year intervals before and after implementation, holding the difference in the quarter immediately before implementation (i.e., “-1” in the figure) at 0. Overall, during the pre-implementation periods (i.e., -10 to -1), almost all estimated differences were not statistically significantly different from zero, and there were no notable trends. These data do not show violations of the parallel trend assumption in the difference-in-differences method.


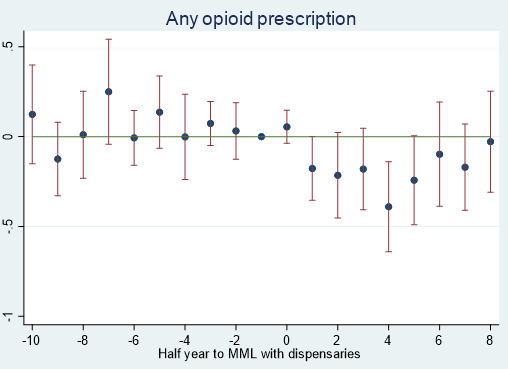

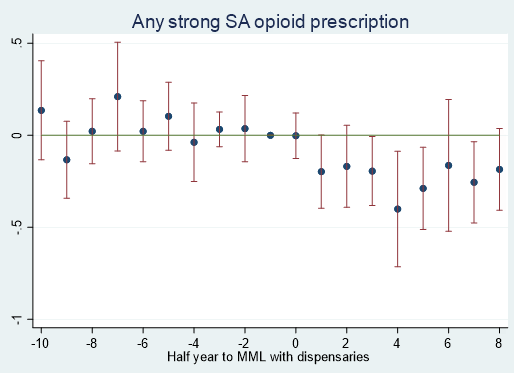

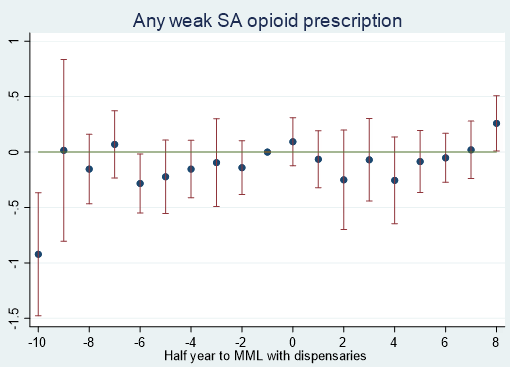


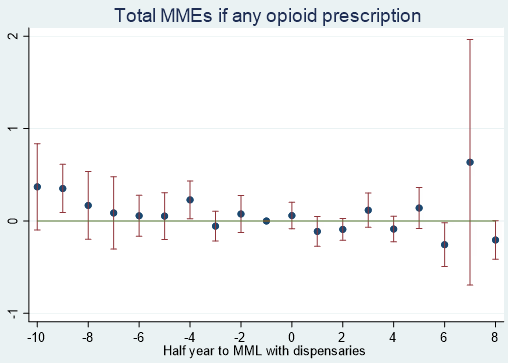

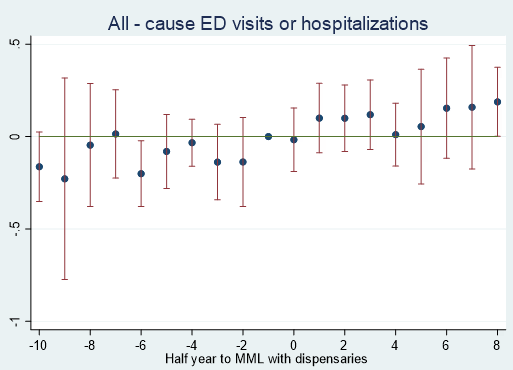

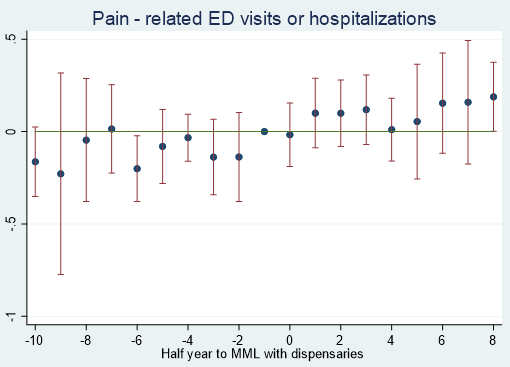


Note: MML=Medical Marijuana Legalization; SA=short-acting; MME=morphine milligram equivalent; ED=emergency department. Strong SA opioids included oxycodone, hydrocodone, hydromorphone, and morphine. Weak SA opioids included tramadol and codeine.

**Appendix 8. Full regression results of the main analysis**

| **Panel A: opioid prescriptions** | **Any opioid prescription (N=34,403)** | | | | **Any strong short-acting opioid prescription (N=34,403)** | | | | **Any weak short-acting opioid prescription (N=34,403)** | | | | **Total MMEs if any opioid prescription (N=18,445)** | | | |
| --- | --- | --- | --- | --- | --- | --- | --- | --- | --- | --- | --- | --- | --- | --- | --- | --- |
|  | Coef. | 95% CI | | p | Coef. | 95% CI | | p | Coef. | 95% CI | | p | Coef. | 95% CI | | p |
| **MML policy (ref=no MML)** |  |  |  |  |  |  |  |  |  |  |  |  |  |  |  |  |
| **MML w/o dispensaries** | -0.055 | -0.195 | 0.084 | 0.438 | -0.080 | -0.218 | 0.057 | 0.249 | **0.134** | **0.052** | **0.216** | **0.002** | -0.129 | -0.310 | 0.052 | 0.153 |
| **MML w/ dispensaries** | -0.207 | -0.435 | 0.022 | 0.072 | **-0.221** | **-0.422** | **-0.020** | **0.028** | 0.119 | -0.033 | 0.271 | 0.135 | -0.136 | -0.400 | 0.127 | 0.292 |
| Cancer type (ref=breast) |  |  |  |  |  |  |  |  |  |  |  |  |  |  |  |  |
| Colorectal | -0.137 | -0.227 | -0.046 | 0.003 | -0.235 | -0.336 | -0.133 | <0.001 | 0.127 | 0.002 | 0.253 | 0.047 | 0.398 | 0.270 | 0.525 | <0.001 |
| Lung | -0.150 | -0.278 | -0.022 | 0.022 | -0.231 | -0.348 | -0.113 | <0.001 | 0.280 | 0.030 | 0.529 | 0.028 | 0.679 | 0.488 | 0.869 | <0.001 |
| Female | -0.055 | -0.134 | 0.025 | 0.18 | -0.109 | -0.201 | -0.016 | 0.022 | 0.103 | -0.054 | 0.260 | 0.199 | -0.297 | -0.461 | -0.134 | <0.001 |
| Age (ref=18-44) |  |  |  |  |  |  |  |  |  |  |  |  |  |  |  |  |
| 45-54 | -0.006 | -0.063 | 0.050 | 0.825 | -0.043 | -0.102 | 0.017 | 0.16 | -0.034 | -0.139 | 0.070 | 0.518 | -0.138 | -0.236 | -0.040 | 0.006 |
| 55-64 | -0.166 | -0.228 | -0.104 | <0.001 | -0.224 | -0.296 | -0.152 | <0.001 | -0.114 | -0.233 | 0.005 | 0.060 | -0.202 | -0.300 | -0.104 | <0.001 |
| Comorbidity |  |  |  |  |  |  |  |  |  |  |  |  |  |  |  |  |
| Back pain | -0.224 | -0.309 | -0.138 | <0.001 | -0.203 | -0.286 | -0.120 | <0.001 | -0.134 | -0.245 | -0.022 | 0.019 | 0.104 | 0.030 | 0.179 | 0.006 |
| Neck pain | -0.081 | -0.146 | -0.016 | 0.014 | -0.086 | -0.153 | -0.018 | 0.013 | 0.010 | -0.162 | 0.182 | 0.909 | -0.066 | -0.140 | 0.008 | 0.080 |
| Arthritis and joint pain | -0.186 | -0.233 | -0.138 | <0.001 | -0.179 | -0.231 | -0.127 | <0.001 | -0.061 | -0.124 | 0.002 | 0.058 | 0.032 | -0.055 | 0.118 | 0.474 |
| Other pain | -0.084 | -0.164 | -0.003 | 0.041 | -0.075 | -0.151 | 0.001 | 0.052 | 0.043 | -0.120 | 0.205 | 0.607 | 0.125 | 0.019 | 0.232 | 0.021 |
| Mental health conditions | -0.004 | -0.077 | 0.069 | 0.913 | 0.011 | -0.052 | 0.075 | 0.731 | 0.053 | -0.055 | 0.161 | 0.334 | 0.147 | 0.065 | 0.228 | <0.001 |
| Alcohol or drug use disorder | -0.228 | -0.369 | -0.086 | 0.002 | -0.185 | -0.311 | -0.060 | 0.004 | -0.184 | -0.445 | 0.076 | 0.166 | 0.258 | -0.192 | 0.708 | 0.261 |
| Nicotine dependence | -0.078 | -0.189 | 0.032 | 0.163 | -0.009 | -0.136 | 0.118 | 0.891 | -0.142 | -0.319 | 0.036 | 0.118 | 0.315 | 0.070 | 0.559 | 0.012 |
| Cancer treatment |  |  |  |  |  |  |  |  |  |  |  |  |  |  |  |  |
| Radiation therapy | -0.012 | -0.070 | 0.046 | 0.688 | -0.060 | -0.134 | 0.014 | 0.111 | -0.018 | -0.102 | 0.067 | 0.682 | -0.063 | -0.108 | -0.018 | 0.007 |
| Chemotherapy | 0.046 | -0.003 | 0.095 | 0.068 | 0.058 | 0.002 | 0.114 | 0.043 | 0.252 | 0.156 | 0.348 | 0.000 | 0.423 | 0.334 | 0.513 | <0.001 |
| Immunotherapy | 0.033 | -0.047 | 0.113 | 0.422 | 0.067 | -0.038 | 0.171 | 0.211 | -0.084 | -0.195 | 0.027 | 0.139 | 0.080 | -0.013 | 0.173 | 0.093 |
| Hormonal therapy | 0.146 | -0.005 | 0.296 | 0.058 | 0.108 | -0.051 | 0.266 | 0.184 | 0.091 | -0.138 | 0.319 | 0.437 | -0.010 | -0.189 | 0.169 | 0.912 |
| Other state policies |  |  |  |  |  |  |  |  |  |  |  |  |  |  |  |  |
| Recreational marijuana legalization | 0.498 | 0.382 | 0.615 | <0.001 | 0.483 | 0.350 | 0.616 | <0.001 | 0.199 | 0.055 | 0.343 | 0.007 | -0.085 | -0.346 | 0.175 | 0.520 |
| Must-access PDMP | 0.033 | -0.154 | 0.221 | 0.727 | -0.078 | -0.251 | 0.095 | 0.376 | -0.025 | -0.155 | 0.105 | 0.708 | -0.068 | -0.266 | 0.131 | 0.503 |
| State legislative limits on opioid quantity or duration | 0.013 | -0.097 | 0.123 | 0.819 | -0.011 | -0.128 | 0.106 | 0.856 | -0.007 | -0.159 | 0.146 | 0.932 | -0.146 | -0.245 | -0.048 | 0.004 |

Note: MML=medical marijuana legalization. MME=morphine milligram equivalents. PDMP=Prescription Drug Monitoring Programs. All models include state and year fixed effects (not reported). Comorbidity includes baseline comorbid conditions during the 12-month look-back period. Cancer treatment includes those received by patients during the 6-month observation period since the new cancer diagnosis.

| **Panel B: adverse hospital events** | **All-cause ED visits or hospitalizations (excluding cancer treatment) (N=34,403)** | | | | **Pain-related ED visits or hospitalizations (N=34,403)** | | | |
| --- | --- | --- | --- | --- | --- | --- | --- | --- |
|  | Coef. | 95% CI | | p | Coef. | 95% CI | | p |
| **MML policy (ref=no MML)** |  |  |  |  |  |  |  |  |
| **MML w/o dispensaries** | 0.067 | 0.001 | 0.134 | 0.050 | 0.112 | -0.073 | 0.297 | 0.241 |
| **MML w/ dispensaries** | **0.166** | **0.050** | **0.282** | **0.006** | 0.165 | -0.146 | 0.476 | 0.315 |
| Cancer type (ref=breast) |  |  |  |  |  |  |  |  |
| Colorectal | 0.390 | 0.326 | 0.455 | <0.001 | 1.179 | 1.053 | 1.304 | <0.001 |
| Lung | 0.424 | 0.270 | 0.578 | <0.001 | 1.497 | 1.340 | 1.654 | <0.001 |
| Female | 0.049 | -0.075 | 0.172 | 0.441 | 0.124 | -0.041 | 0.289 | 0.142 |
| Age (ref=18-44) |  |  |  |  |  |  |  |  |
| 45-54 | -0.111 | -0.200 | -0.021 | 0.015 | -0.162 | -0.277 | -0.047 | 0.006 |
| 55-64 | -0.198 | -0.292 | -0.103 | <0.001 | -0.421 | -0.546 | -0.297 | <0.001 |
| Comorbidity |  |  |  |  |  |  |  |  |
| Back pain | 0.171 | 0.103 | 0.239 | <0.001 | 0.368 | 0.254 | 0.482 | <0.001 |
| Neck pain | -0.035 | -0.138 | 0.069 | 0.513 | 0.046 | -0.136 | 0.228 | 0.622 |
| Arthritis and joint pain | 0.125 | 0.068 | 0.181 | <0.001 | 0.170 | 0.075 | 0.264 | <0.001 |
| Other pain | 0.331 | 0.266 | 0.397 | <0.001 | 0.405 | 0.280 | 0.530 | <0.001 |
| Mental health conditions | 0.264 | 0.205 | 0.322 | <0.001 | 0.232 | 0.139 | 0.325 | <0.001 |
| Alcohol or drug use disorder | 0.225 | 0.042 | 0.408 | 0.016 | 0.425 | 0.168 | 0.683 | 0.001 |
| Nicotine dependence | 0.285 | 0.145 | 0.426 | <0.001 | 0.292 | 0.124 | 0.460 | 0.001 |
| Cancer treatment |  |  |  |  |  |  |  |  |
| Radiation therapy | -0.282 | -0.368 | -0.197 | <0.001 | -0.099 | -0.204 | 0.006 | 0.064 |
| Chemotherapy | 0.790 | 0.698 | 0.882 | <0.001 | 0.569 | 0.477 | 0.662 | <0.001 |
| Immunotherapy | 0.164 | 0.073 | 0.254 | <0.001 | 0.190 | 0.061 | 0.319 | 0.004 |
| Hormonal therapy | -0.163 | -0.300 | -0.026 | 0.020 | -0.138 | -0.380 | 0.104 | 0.264 |
| Other state policies |  |  |  |  |  |  |  |  |
| Recreational marijuana legalization | -0.352 | -0.493 | -0.211 | <0.001 | 0.126 | -0.138 | 0.389 | 0.350 |
| Must-access PDMP | -0.036 | -0.140 | 0.067 | 0.490 | 0.064 | -0.146 | 0.275 | 0.549 |
| State legislative limits on opioid quantity or duration | -0.004 | -0.095 | 0.088 | 0.933 | -0.069 | -0.233 | 0.094 | 0.408 |

Note: MML=medical marijuana legalization. MME=morphine milligram equivalents. PDMP=Prescription Drug Monitoring Programs. ED= emergency department. All models include state and year fixed effects (not reported). Comorbidity includes baseline comorbid conditions during the 12-month look-back period. Cancer treatment includes those received by patients during the 6-month observation period since the new cancer diagnosis.

**Appendix 9. de Chaisemartin and d’Haultfoeuille difference-in-differences model and supplemental analysis results**

Model specification

The method developed by de Chaisemartin and d’Haultfoeuille is designed to address possible heterogeneous treatment effects in the difference-in-differences design (i.e., the fact that policy impacts may vary across states and over time). In the context of our study, this means that the effects of medical marijuana legalization (MML) policies, if any, may differ by state or by how long the policy has been in effect. See de Chaisemartin and d’Haultfoeuille (2023) for details: de Chaisemartin C, Haultfœuille X. Two-way fixed effects and differences-in-differences estimators with several treatments. Journal of Econometrics. 2023;236(2):105480. doi:10.1016/j.jeconom.2023.105480

Notably, de Chaisemartin and d’Haultfoeuille method examines one dichotomous policy indicator at a time, and assumes that once a policy is implemented, it always stays on. Based on this framework and the policy landscape of MML policies (see Appendix 1), we defined the following two policy indicators and estimated the effects of each in separate regression models. Note that these policy indicators are different from those used in our main analysis. The resulting policy comparators are also different but still useful in assessing the robustness of the results of our main analysis.

- **MML:** This indicator is coded as 1 if a state has implemented MML, regardless of whether dispensaries are available, and 0 otherwise (i.e., 1=MML, including MML with and without dispensaries; 0=no MML).
- **MML with dispensary:** This indicator is coded as 1 if a state has implemented MML and at least one medical dispensary is open, and 0 otherwise (i.e., 1=MML with dispensaries; 0=no MML or MML without dispensaries).

We used the “**did_multiplegt_dyn**” package in Stata for this supplemental analysis. All models control for the same set of covariates in the main analysis, including patient sex, age, comorbid conditions associated with needs for and/or risks of opioid analgesics (back pain, neck pain, arthritis and joint pain, other pain, mental health conditions, alcohol or drug use disorder, and nicotine dependence), and cancer treatment other than resection surgery during the 6-month observation period (radiation therapy, chemotherapy, immunotherapy, and hormonal therapy). We clustered standard errors at the state level since MML policies are implemented at the state level. A p-value < 0.05 indicates statistical significance.

Results of supplemental analysis

| **Policy effect: MML** (vs no MML) | **de Chaisemartin and d’Haultfoeuille method** | | | |
| --- | --- | --- | --- | --- |
|  | **Estimate** | **95% CI** | | **p-value** |
| Any opioid prescription | -0.004 | -0.030 | 0.021 | 0.755 |
| Any strong short-acting opioid prescription | -0.020 | -0.041 | 0.001 | 0.058 |
| Any weak short-acting opioid prescription | 0.013* | 0.002 | 0.024 | 0.021 |
| All-cause ED visits or hospitalizations | 0.021* | 0.007 | 0.035 | 0.003 |
| Pain-related ED visits or hospitalizations | 0.025* | 0.015 | 0.036 | <0.001 |

Note: MML=Medical Marijuana Legalization; MME=morphine milligram equivalent; ED=emergency department. Strong short-acting opioids included oxycodone, hydrocodone, hydromorphone, and morphine. Weak short-acting opioids included tramadol and codeine. *indicates statistically significant results at 0.05 level.

| **Policy effect: MML with dispensaries**  (vs no MML and MML without dispensaries) | **de Chaisemartin and d’Haultfoeuille method** | | | |
| --- | --- | --- | --- | --- |
|  | **Estimate** | **95% CI** | | **p-value** |
| Any opioid prescription | -0.045* | -0.076 | -0.014 | 0.005 |
| Any strong short-acting opioid prescription | -0.041* | -0.063 | -0.018 | <0.001 |
| Any weak short-acting opioid prescription | -0.022* | -0.033 | -0.011 | <0.001 |
| All-cause ED visits or hospitalizations | 0.032* | 0.017 | 0.046 | <0.001 |
| Pain-related ED visits or hospitalizations | 0.018* | 0.008 | 0.028 | <0.001 |

Note: MML=Medical Marijuana Legalization; MME=morphine milligram equivalent; ED=emergency department. Strong short-acting opioids included oxycodone, hydrocodone, hydromorphone, and morphine. Weak short-acting opioids included tramadol and codeine. *indicates statistically significant results at 0.05 level.

**Appendix 10. Distribution of days from cancer diagnosis to resection surgery**

| **Days from cancer diagnosis to resection surgery** | **N** | **%** | **Cumulative %** |
| --- | --- | --- | --- |
| <0 (and within the same month of cancer diagnosis) | <800 | 0.72 | 0.72 |
| 0-30 | 15249 | 43.68 | 44.4 |
| 31-60 | 12175 | 34.87 | 79.28 |
| 61-90 | 3064 | 8.78 | 88.05 |
| 91-120 | 1109 | 3.18 | 91.23 |
| 121-150 | 1464 | 4.19 | 95.42 |
| >150 | 1598 | 4.58 | 100 |

**Appendix 11. Sample characteristics and unadjusted outcomes by cancer type**

|  | **By Cancer Type** | | | | | |
| --- | --- | --- | --- | --- | --- | --- |
|  | **Breast** | | **Colorectal** | | **Lung** | |
| N | 24,592 | | 8,510 | | 1,809 | |
| Sample Characteristics | N | % | N | % | N | % |
| Female | 24,592 | 100.0% | 3,568 | 41.9% | 1,009 | 55.8% |
| Age |  |  |  |  |  |  |
| 18-44 | 3,920 | 15.9% | 1,114 | 13.1% | <800 | n/a |
| 45-54 | 8,955 | 36.4% | 3,235 | 38.0% | <800 | n/a |
| 55-64 | 11,717 | 47.7% | 4,161 | 48.9% | 1,396 | 77.2% |
| Comorbidity |  |  |  |  |  |  |
| Back pain | 3,999 | 16.3% | 1,186 | 13.9% | <800 | n/a |
| Neck pain | 2,217 | 9.0% | <800 | n/a | <800 | n/a |
| Arthritis and joint pain | 5,932 | 24.1% | 1,508 | 17.7% | <800 | n/a |
| Other pain | 5,141 | 20.9% | 1,791 | 21.1% | <800 | n/a |
| Mental health conditions | 5,737 | 23.3% | 1,329 | 15.6% | <800 | n/a |
| Alcohol or drug use disorder | <800 | n/a | <800 | n/a | <800 | n/a |
| Nicotine dependence | <800 | n/a | <800 | n/a | <800 | n/a |
| Cancer treatment (during the 6-month observation period since the new cancer diagnosis) |  |  |  |  |  |  |
| Radiation therapy | 12,718 | 51.7% | 1,561 | 18.3% | <800 | n/a |
| Chemotherapy | 9,844 | 40.0% | 4,273 | 50.2% | <800 | n/a |
| Immunotherapy | 2,958 | 9.2% | <800 | n/a | <800 | n/a |
| Hormonal therapy | <800 | n/a | <800 | n/a | <800 | n/a |
| Unadjusted Outcomes |  |  |  |  |  |  |
| Opioid prescriptions |  |  |  |  |  |  |
| Any opioid prescription | 9,957 | 40.5% | 3,274 | 38.5% | <800 | n/a |
| Any strong short-acting opioid prescription (i.e., oxycodone, hydrocodone, hydromorphone, or morphine) | 8,521 | 34.7% | 2,720 | 32.0% | <800 | n/a |
| Any weak short-acting opioid prescription (i.e., tramadol or codeine) | 2,514 | 10.2% | 931 | 10.9% | <800 | n/a |
| Total MMEs if any opioid prescription, mean (SD) | 309.82  (422.75) | | 656.19  (2368.08) | | 811.09  (1558.54) | |
| Adverse hospital events |  |  |  |  |  |  |
| All-cause ED visits or hospitalizations (excluding cancer treatment) | 4,380 | 17.8% | 2,193 | 25.8% | <800 | n/a |
| Pain-related ED visits or hospitalizations | <800 | n/a | <800 | n/a | <800 | n/a |

Cells with <800 patients contributing to the estimates were suppressed to comply with the Health Care Cost Institute Reporting Guidelines.

Note: MME=morphine milligram equivalent; ED=emergency department

**Appendix 12. Estimates of medical marijuana policy effects on outcomes by cancer type**

| **Policy effect** | **MML w/o dispensaries (vs no MML)** | | | | **MML w/ dispensaries (vs no MML)** | | | | **Incremental effects of dispensaries  ( MML w/ dispensaries vs MML w/o dispensaries)** | | | |
| --- | --- | --- | --- | --- | --- | --- | --- | --- | --- | --- | --- | --- |
| **Breast cancer (N=24,592)** | **Estimate** | **95% CI** | | **p** | **Estimate** | **95% CI** | | **p** | **Estimate** | **95% CI** | | **p** |
| Any opioid prescription | -0.008 | -0.047 | 0.031 | 0.687 | -0.050 | -0.102 | 0.003 | 0.064 | -0.042 | -0.090 | 0.006 | 0.089 |
| Any strong short-acting opioid prescription | -0.011 | -0.043 | 0.020 | 0.483 | -0.048* | -0.088 | -0.009 | 0.016 | -0.037 | -0.081 | 0.007 | 0.098 |
| Any weak short-acting opioid prescription | 0.016* | 0.001 | 0.031 | 0.035 | 0.015 | -0.002 | 0.032 | 0.080 | -0.001 | -0.018 | 0.016 | 0.932 |
| Total MMEs if any opioid prescription | -13.18 | -51.63 | 25.28 | 0.502 | -15.29 | -58.46 | 27.87 | 0.487 | -2.12 | -34.00 | 29.77 | 0.897 |
| All-cause ED visits or hospitalizations | 0.004 | -0.012 | 0.020 | 0.605 | 0.022* | 0.001 | 0.043 | 0.040 | 0.018* | 0.005 | 0.031 | 0.007 |
| Pain-related ED visits or hospitalizations | 0.000 | -0.011 | 0.010 | 0.969 | -0.001 | -0.013 | 0.011 | 0.889 | -0.001 | -0.009 | 0.008 | 0.875 |
| **Colorectal cancer (N=8,510)** | **Estimate** | **95% CI** | | **p** | **Estimate** | **95% CI** | | **p** | **Estimate** | **95% CI** | | **P** |
| Any opioid prescription | -0.018 | -0.070 | 0.035 | 0.507 | -0.039 | -0.105 | 0.027 | 0.249 | -0.021 | -0.054 | 0.011 | 0.204 |
| Any strong short-acting opioid prescription | -0.020 | -0.069 | 0.028 | 0.411 | -0.035 | -0.098 | 0.027 | 0.268 | -0.015 | -0.051 | 0.021 | 0.406 |
| Any weak short-acting opioid prescription | 0.000 | -0.023 | 0.023 | 0.972 | -0.005 | -0.042 | 0.033 | 0.796 | -0.005 | -0.025 | 0.016 | 0.670 |
| Total MMEs if any opioid prescription | -181.26* | -319.44 | -43.07 | 0.010 | -164.93 | -375.80 | 45.94 | 0.125 | 16.32 | -115.62 | 148.27 | 0.808 |
| All-cause ED visits or hospitalizations | 0.034 | -0.004 | 0.072 | 0.076 | 0.051* | 0.000 | 0.102 | 0.049 | 0.017 | -0.015 | 0.049 | 0.291 |
| Pain-related ED visits or hospitalizations | 0.032 | -0.004 | 0.068 | 0.077 | 0.041 | -0.013 | 0.096 | 0.132 | 0.009 | -0.024 | 0.042 | 0.582 |
| **Lung cancer (N=1,809)** | **Estimate** | **95% CI** | | **p** | **Estimate** | **95% CI** | | **p** | **Estimate** | **95% CI** | | **p** |
| Any opioid prescription | -0.033 | -0.106 | 0.039 | 0.365 | -0.040 | -0.101 | 0.020 | 0.191 | -0.007 | -0.062 | 0.047 | 0.799 |
| Any strong short-acting opioid prescription | -0.048 | -0.125 | 0.029 | 0.220 | -0.028 | -0.090 | 0.034 | 0.379 | 0.020 | -0.038 | 0.079 | 0.491 |
| Any weak short-acting opioid prescription | 0.036 | -0.032 | 0.104 | 0.305 | 0.006 | -0.042 | 0.053 | 0.819 | -0.030 | -0.070 | 0.010 | 0.143 |
| Total MMEs if any opioid prescription | 122.14 | -477.65 | 721.93 | 0.690 | -179.01 | -483.10 | 125.09 | 0.249 | -301.15 | -600.18 | 57.89 | 0.100 |
| All-cause ED visits or hospitalizations | -0.026 | -0.127 | 0.075 | 0.610 | -0.030 | -0.160 | 0.099 | 0.644 | -0.004 | -0.125 | 0.117 | 0.946 |
| Pain-related ED visits or hospitalizations | -0.035 | -0.107 | 0.037 | 0.338 | -0.009 | -0.107 | 0.089 | 0.855 | 0.026 | -0.044 | 0.096 | 0.466 |

Note: MML=Medical Marijuana Legalization; MME=morphine milligram equivalent; ED=emergency department. Strong short-acting opioids included oxycodone, hydrocodone, hydromorphone, and morphine. Weak short-acting opioids included tramadol and codeine. *indicates statistically significant results at 0.05 level.

**Appendix 13. Predicted probabilities of opioid prescription outcomes associated with different medical marijuana policies by cancer type**

1. Any opioid prescription
2. Any strong short-acting opioid prescription
3. Any weak short-acting opioid prescription

**Appendix 14. Estimated total MMEs associated with different medical marijuana policies among patients any opioid prescription by cancer type**

**Appendix 15. Predicted probabilities of adverse hospital** **events associated with different medical marijuana policies by cancer type**
